# Influence of Backbone Ladderization and Side Chain Variation on the Orientation of Diketopyrrolopyrrole-Based Donor-Acceptor Copolymers

**DOI:** 10.3390/molecules28186435

**Published:** 2023-09-05

**Authors:** Sven Bölke, Andreas Früh, Florian Trilling, Michael Forster, Ullrich Scherf, Thomas Chassé, Heiko Peisert

**Affiliations:** 1 Institut für Physikalische und Theoretische Chemie, Eberhard Karls Universität Tübingen, Auf der Morgenstelle 18, 72076 Tübingen, Germanyfrueh.andreas@gmail.com (A.F.);; 2 Makromolekulare Chemie (*buwMakro*) und Wuppertal Center for Smart Materials and Systems (CM@S), Bergische Universität Wuppertal, Gaussstrasse 20, 42119 Wuppertal, Germanymforster@uni-wuppertal.de (M.F.);

**Keywords:** low band gap polymers, orientation, UV/vis, FTIR, PM-IRRAS, benzodithiophene, benzothiadiazole

## Abstract

Ladder polymers with poly(diketopyrrolopyrrole) (DPP) moieties have recently attracted enormous interest for a large variety of opto-electronic applications. Since the rigidity of the backbone increases with ladderization, a strong influence on the self-organization of thin films is expected. We study the molecular orientation of DPP-based ladder polymers in about 50 nm thin films using polarization modulation-infrared reflection-absorption spectroscopy (PM-IRRAS). Exemplarily, for one polymer, the orientation in thicker films is qualitatively investigated by infrared spectroscopy in transmission. Further, this method allows us to rule out the effects of a possible azimuthal ordering, which would affect the analysis of the orientation by PM-IRRAS. For all polymers, the long axis of the polymer backbone is preferentially oriented parallel to the substrate surface, pointing to a high degree of ordering. It is suggested that the choice of the side chains might be a promising way to tune for face-on and edge-on orientations. The exemplarily performed investigation of interface properties on substrates with different work functions suggests that the choice of the side chains has a minor effect on the interfacial electronic interface structure.

## 1. Introduction

Poly(diketopyrrolopyrrole) (DPP)-based ladder polymers (LPs) have attracted enormous interest in a broad field of applications. For cancer therapy and imaging, properties such as high thermal and photochemical stability, efficient generation of reactive oxygen species, easy functionalization, and tunable photophysical properties are of particular interest [1]. The large variety of opto-electronic applications includes solar cells, field-effect transistors, and thermoelectric devices [2,3,4,5,6,7]. Cyano-substituted benzochalcogenadiazole and DPP allow the development of high-efficiency n-type organic thermoelectrics [8]. An exceptionally high charge mobility for high-performance electronics was recently observed [9]. In addition, they have the potential to reduce the energy consumption and costs of industrial chemical separations [10] and can be highly enantioselective [11]. Even polymer lasers and all-optical devices based on the principle of strong light–matter coupling are possible and under investigation [12].

It is well known that the properties of low band gap (LBG) polymers in devices are strongly influenced by their morphology and ability for self-organization in thin films. In the case of LP, beside the kind and position of side chains, the degree of ladderization can also strongly affect the aggregation in thin films [13,14,15]. The crystallinity and the detailed molecular orientation of thin films can substantially affect the field-effect electron mobility [16]. The tuning of aggregation properties allows the increase of the open circuit voltage and short-circuit current density in organic solar cells, resulting in an enhancement of the power conversion efficiency [7].

**Figure 1:**
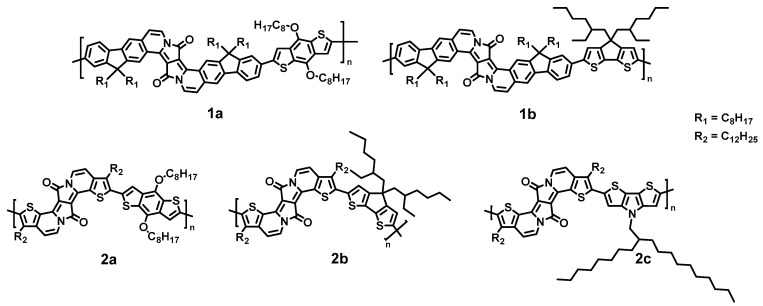
Chemical structures of investigated DPP-based step-ladder copolymers with different backbones (1 vs. 2) and side chains (a vs. b vs. c). Note, that also due to the different backbone structure, side chains a and b are not exactly the same.

We study the molecular orientation of DPP-based donor-acceptor copolymers, denoted as 1a, 1b, 2a, 2b, and 2c (the number refers to the DPP-unit size and the letter refers to the type of the thiophene-extended unit) (Figure 1). We use infrared (IR) spectroscopy for the determination of the molecular orientation in thin films. Principally, the method can be applied through reflection or transmission. Since IR spectroscopy is available in many laboratories, this method might be increasingly applied in the future for orientation studies. In the case of infrared reflection absorption spectroscopy (IRRAS, also called RAIRS: reflection–absorption infrared spectroscopy), the surface sensitivity and signal/noise ratio can be distinctly increased if polarized IR radiation is modulated between parallel (p-) and perpendicular (s-) polarization with respect to the incident reflection plane [17]. This method is called polarization modulation infrared reflection absorption spectroscopy (PM-IRRAS). For the determination of the molecular orientation in (ultra) thin films, IRRAS utilizes the surface selection rule, causing a drastic suppression of molecular vibrations with an oscillating dipole moment parallel to the (metallic) surface (e.g., Refs. [18,19,20,21]). The angle of incidence of the IR radiation is typically close to grazing incidence (ca. ~80°), which also delivers the maximal intensity. PM-IRRAS is used in the present study for the determination of the orientation of the molecular backbone of polymers shown in Figure 1. Additional investigations were performed by IR spectroscopy in transmission.

## 2. Results and Discussion

### 2.1. Choice of Polymers and Characterization

**Figure 2:**
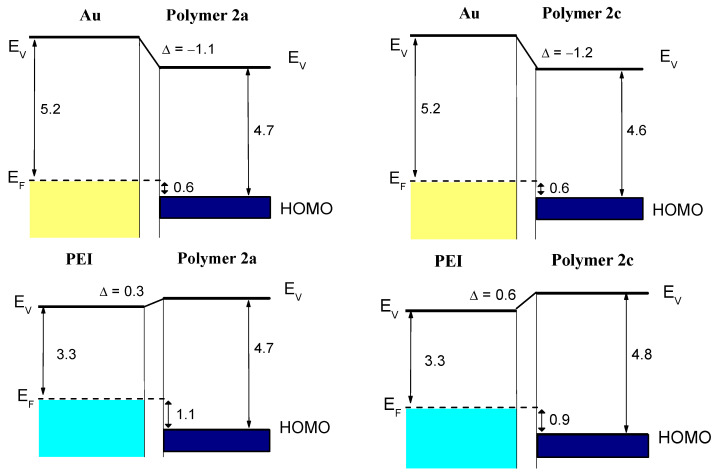
Schematic energy level diagrams of polymers 2a and 2c on Au and PEI. All values given in eV. The pinning positions (difference between the HOMO energy and the Fermi level E_F_ of the substrate) are similar for both polymers on high- or low-work-function materials. Such pinning positions can be related to positive or negative polaron levels.

The DPP polymers 2a, 2b, and 2c (Figure 1) possess an extended π-system (with thiophen), so-called π-expanded DPPs (EDPPT); polymers 1a and 1b are structurally based on fluorene-extended EDPP derivatives. Side chains (-alkyl and -alkoxy) were varied. The choice of these structures was motivated by the crucial role of ladderization in the ability of self-organization in thin films as well as the reduced energy gaps between the highest occupied orbital (HOMO) and the lowest unoccupied orbital (LUMO) in the case of thiophene-extended units [22]. The optical band gap (E_g_) is about 2.1 eV for polymers 1 and 1b and 1.6–1.7 eV for polymers 2 [22]. Exemplarily for polymers 2a and 2c, interface properties on the high work function (Φ) material gold (Φ = 5.2 ± 0.1 eV) and the low work function material polyethylenimine (PEI, Φ = 3.3 eV) were investigated by ultraviolet photoelectron spectroscopy (UPS). The resulting energy level alignment diagrams are shown in Figure 2, and the related UPS spectra in Figure S1 (Supplementary Materials). The ionization potential (IP), i.e., the energy separation between the vacuum level and the HOMO onset, is 4.7 ± 0.1 eV and thus typical for p-type materials (cf. Refs. [23,24,25]). Clearly visible in Figure 2, the energy level alignment at the studied interfaces does not follow a vacuum level alignment regime; dipoles are formed in all cases, and a so-called “pinning” of energy levels is observed. For gold, the pinning level on gold is found at 0.6 eV (Figure 2) and on PEI at 1.1 and 0.9 eV for polymers 2a and 2c, respectively. In the frame of the integer charge transfer (ICT) model [26,27,28], such pinning positions can be interpreted as the pinning at geometrically fully relaxed positive (negative) polaron levels. In the case of a high work function material (gold), an electron is transferred from the HOMO to the substrate, and a level is formed called the positive integer charge transfer level ICT^+^. In contrast, in the case of a low-work-function material, a charge transfer in the opposite direction takes place, and the negative ICT^−^ is formed. These charge transfer levels are relevant for transport properties. They are similar for polymers 2a and 2c, indicating that the variation of the side chain has only small effects on the electronic (interface) properties.

### 2.2. Molecular Orientation of Polymers in Thin Films Determined by PM-IRRAS

Infrared spectroscopy in reflection, and in particular PM-IRRAS, is meanwhile well-established for the determination of the molecular orientation, especially for ultrathin films ranging from monolayer coverages to 50–100 nm (e.g., Refs. [20,29,30,31,32,33]). For thicker films, the film thickness has to be considered for a quantitative evaluation of the data [34]. For the relative data analysis method (as used here), a reference with random orientation (i.e., an average of all possible orientations) is used. In our case, a well-ground polymer powder was pressed into KBr.

**Figure 3:**
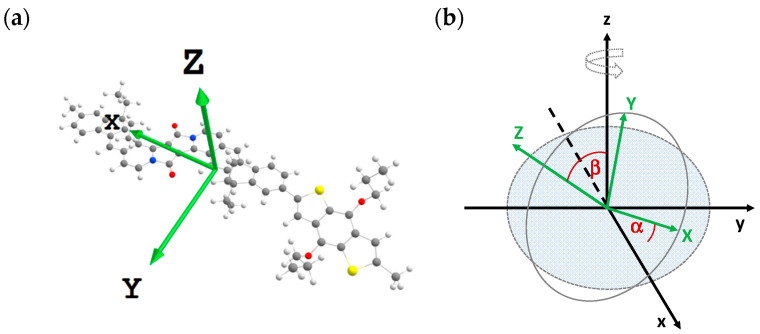
Definition of (**a**) the molecules’ internal coordinate system XYZ (green) and (**b**) the orientation of XYZ in a three-dimensional room xyz described with the angles α and β, where xy can be regarded as substrate plane.

First, we define the two coordinate systems used in the following discussion of molecular orientation (Figure 3). The first one is a three-dimensional XYZ-system related to the molecule, where in the XY-plane the polymer backbone is situated. The Z-vector is orthogonal to this plane (see Figure 3a, capital letters). We note that a small bending in the molecular structure is not considered. The second xyz-coordinate system (lowercase letters) is related to the substrate; the xy-plane corresponds to the substrate surface (Figure 3b). The angle between X and the xy plane is defined as α and the angle between Z and the surface normal z as β.

For the data analysis, an accurate assignment of the vibration bands is essential. The results of density-functional theory (DFT) calculations are summarized in Table 1. For the data analysis, we use qualified vibration bands with dipole moments lying in the molecular plane XY as well as pointing out of this plane (Z): (1) the C=O stretching vibration at about 1680 cm^−1^ (Y), (2) the CN/CC stretching vibration at 1626 cm^−1^ (X) and (3) the CH out-of-plan vibration at 822/780 cm^−1^ (Z). The direction of dipole moments of these vibrations derived from DFT calculations (green arrows in Figure 3a) is in good agreement with the cartesian coordinates (deviations less than 5°).

**Figure 4:**
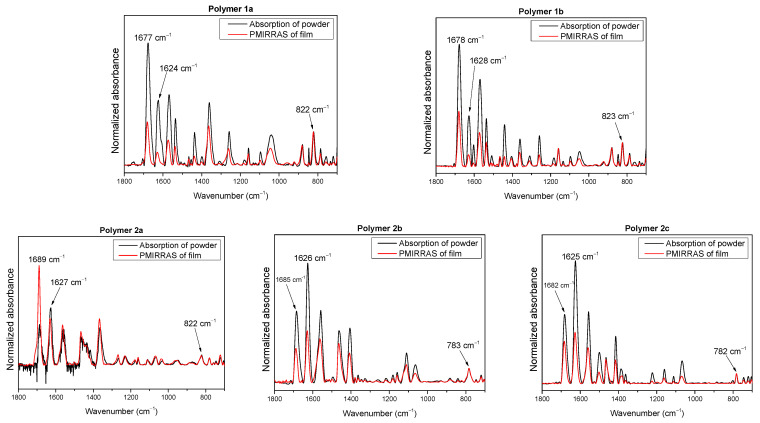
PM-IRRAS spectra of KBr pellets (black) and thin films (red) of polymers 1 and 2 with wavenumbers of bands used for determination of the molecular orientation.

In Figure 4, PM-IRRAS spectra for all five polymers on gold are shown (red curves) together with reference spectra (powder in KBr). The film thickness was less than 100 nm in all cases. The red spectra in Figure 4 were normalized to the band of powder at 822 cm^−1^ and 782 cm^−1^, respectively.

Very strong intensity deviations between the red and black curves are clearly visible in Figure 4, indicating a preferred orientation of the polymer in film. From the lower intensities for the film compared to the powder for the band at around 1626 cm^−1^ (considering the selection rules and the direction of the dipole moment of this band parallel to the X-axis), one can already conclude a preferred parallel orientation of the polymer backbone with respect to the substrate surface (“face-on” or “edge-on”).

Following the approach of Debe [35], we calculated the Euler angles according to ${sin}^{2}\Psi =\frac{1}{1+r(zx)}$ and ${sin}^{2}\Theta =\frac{1+r(zx)}{1+r\left(yx\right)+r(zx)}$ where *x*, *y* and *z* are the intensities of the above-defined IR signals and *r* is the ratio between these intensities with respect to the reference (pellet). The angles α and β between the polymer backbone and the sample surface were obtained by using a rotation matrix (cf. Ref. [21]). We note that this approximation includes an azimuthal averaging of molecular orientations.

The angles α and β are summarized in Table 2 for all studied polymers. We note that these angles denote “average angles”, i.e., the angular distribution or the presence of partly disordered regions is neglected. The angle α is found for all polymers in the range between 21° and 28°. In other words, all investigated polymers show a clear trend toward a parallel orientation of the “long axis” (X) of the backbone with respect to the substrate surface. Such a preferred molecular orientation indicates a high degree of ordering and self-organization and depends crucially on the chemical structure and preparation conditions (e.g., Refs. [36,37,38]). Remarkably, α is distinctly smaller for polymers 1 compared to polymers 2. Thus, it seems that the higher degree of ladderization supports such a preferred parallel orientation of the backbone relative to the substrate, independent of the different side chains of polymers 1a and 1b. The angle β describes the orientation of the “short axis” (Y) of the polymer backbone with respect to the substrate surface. In this manner, an “edge-on” orientation (β = 90°) can be distinguished from “face-on” (β = 0°). The angle β is very similar for all polymers except 2a (Table 2). Values between 29° and 33° indicate a preferred face-on orientation. The higher value of β = 50° for polymer 2a might be caused by the nature of the side chain. For related LPs, it was reported that polymers with linear alkoxy side chains tend to arrange in an edge-on geometry [13] or a mixture of face-on and edge-on [14]—in contrast to polymers with bulky branched alkyl chains (like 2b and 2c). Thus, the tuning of the side chains might be a promising way to control face-on and edge-on orientations.

### 2.3. Azimuthal Orientation and Thickness-Dependence Studied Using IR Spectroscopy in Transmission

IR spectroscopy in transmission can be principally applied for the determination of the orientation of thin films since the selection rules are mutual (orthogonal) compared to IRRAS. For a transmission experiment, the electric field vector of the IR light is parallel to the substrate surface and perpendicular in the case of IRRAS. However, the quantitative analysis of transmission spectra includes the consideration of many optical parameters involving complex refractive index as a function of wavenumber and thickness [39,40]. Most difficulties can be overcome by measurements in a selected angle region, depending on the considered sample (multiple-angle incidence resolution spectrometry, MAIRS) [39,40]. Alternatively, thicker films (in the µm range) were measured at different angles of the incoming IR light [41]. In the following, we will discuss results from IR experiments in transmission (qualitatively only).

**Figure 5:**
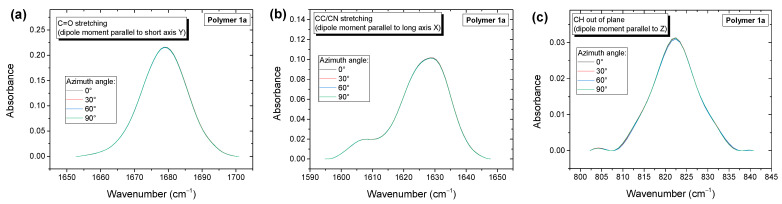
IR spectra in transmission (polar angle 0°) of an approx. 1200 nm thick film of polymer 1a. (**a**) C=O stretching vibration; (**b**) CN/CC stretching vibration (**c**) CH out-of-plan vibration. The absolute absorbance does not depend on the polarization (azimuth) of the linearly polarized light, indicating the uniaxial distribution of molecular orientation.

Since the applied preparation method for the thin films was doctor blade casing (i.e., a blade is moved in a well-defined manner over the substrate surface), a preferred azimuthal orientation of the polymer backbones parallel to the moving direction of the blade might be expected. On the other hand, azimuthal averaging is a precondition for the analysis of PM-IRRAS data, as performed in Section 2.1. Therefore, IR experiments in transition with linearly polarized light were performed, as exemplarily shown for polymer 1a in Figure 5. The azimuthal angle of the incoming IR light was varied from 0° to 90° to monitor possible changes in the molecular orientation, which would cause a variation in the IR intensities. The azimuth angle of 90° corresponds to the electric field vector parallel to the moving direction of the doctor’s blade. In Figure 5, we focus on the three wavelength regions of vibration bands used for the determination of the orientation in Section 2.1, spectra for the other polymers are shown in the Supplementary Materials (Figures S2–S5). For all three regions in Figure 5, the spectra taken at different azimuth angles are almost superimposable; differences in the (absolute) absorbance are less than 1%. In other words, the existence of a preferred azimuthal orientation can be excluded. We note that for the other polymers, differences in the absorbance were less than 3%, confirming a uniaxial distribution along the z-axis (Figures S2–S5, Supplementary Materials).

Further, the question may arise whether or not the molecular orientation of the polymers determined by PM-IRRAS (Section 2.1) is changed if the film thickness increases. The absence of any thickness dependence may indicate that the orientation in the 50 nm thin films is not noticeably affected by the substrate surface but rather driven by the self-organization in the film. Since the relative method of the IRRAS technique is limited to thicknesses of about 100 nm [21], we used IR spectroscopy in transmission to qualitatively evaluate the orientation of thicker films. To avoid the aforementioned complications of transmission measurements at different incidence angles of the incoming IR light, all measurements were performed at normal incidence.

**Figure 6:**
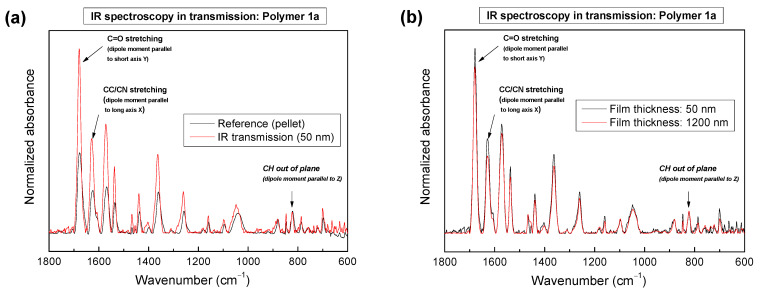
IR spectra in transmission (normal incidence, polymer 1a). (**a**) Comparison of the transmission spectrum to the reference of a randomly ordered sample (powder pressed in KBr). (**b**) Comparison of transmission spectra for two significantly different thicknesses (50 nm and 1200 nm). All spectra are normalized to the out-of-plane CH vibration at 822 cm^−1^.

In Figure 6a, we compare the IR spectrum of a 50 nm thick film of polymer 1a measured in transmission geometry with the powder spectrum in KBr (reference with random orientation). Clearly visible, vibrations with transition dipole moments in the X and Y directions (molecule coordinates) are more intense in the film with respect to the random reference (KBr pellet) when normalized to the out-of-plane CH vibration at 822 cm^−1^. Due to the orthogonal selection rules, vibrations in the plane of the substrate surface in transmission geometry are selectively excited and not attenuated as observed in PM-IRRAS (cf. Figure 4). Thus, qualitatively, a preferred face-on orientation parallel to the substrate surface can be concluded for a 50 nm thick film of polymer 1a.

Also, for a film thickness of 1200 nm, an enhancement of in-plane vibrations with respect to the reference is observed, resulting in a preferred face-on orientation. A direct comparison of transmission spectra for different thicknesses in Figure 6b reveals only a small decrease of the in-plane vibrations (C=O and CC/CN with respect to CH oop) of about 15% (maximum of the intensity) for the very thick 1200 nm film compared to a film thickness of 50 nm. Thus, the dependence of the orientation on the thickness is very weak for polymer 1a. We note that the orientation of thicker films of polymers 1b, 2a, 2b, and 2c, as qualitatively determined from transmission experiments, is in good agreement with PM-IRRAS data of 50 nm films (Supplementary Materials, Figures S6–S9).

## 3. Materials and Methods

### 3.1. Materials and Sample Preparation

The polymers were synthesized as recently reported [22]. The measured molecular weight distributions are summarized in Table 3.

Polymer thin films were prepared at room temperature by doctor-blade casting in a nitrogen atmosphere from 0.5% to 2% (wt) solutions in chloroform. The blade distance to the substrate surface was 350 µm for all samples. The coating thicknesses were further controlled by the speed of the blade in the range between 10 mm/s and 40 mm/s. As substrates, gold-covered silicon wafers and KBr (Korth Kristalle, Altenholz, Germany) were used for PM-IRRAS and IR transmission, respectively. All substrates were cleaned using iso-propanol and chloroform, followed by a UV/ozone treatment (SEN LIGHTS Corp., Osaka, Japan, Photo Surface Processor PL16-110B-1) for 5–15 min. The gold substrates for the UPS measurements were treated with UV/ozone for 1 h. PEI substrates were obtained by doctor-blade casting of polyethylenimine (0.1% (*w*/*w*) solution polyethylenimine in isobutanol) in indium-tin-oxide (ITO, Hoya Corporation, Tokyo, Japan, sheet resistance R = 10 Ω/□) at 80 °C and subsequent annealing to 110 °C for 10 min.

### 3.2. Methods

For the IR transmission and PM-IRRAS measurements, a Vertex 70v spectrometer (Bruker, Billerica, MA, USA) with a PMA50 module was used. A polarizer (K276, Optomatrics Corp., Littleton, CO, USA) was used for the investigations using polarized light (azimuthal orientation, IR in transmission). The UPS measurements were performed in a multichamber ultra-high vacuum system equipped with an Omicron hemispherical analyzer (Omicron Vakuumphysik, Taunusstein, Germany, EA 125) and a helium discharge lamp (Leybold–Heraeus, Köln, Germany, UVS10/35) using He I radiation (21.22 eV).

The DFT calculations for the trimers and shortened (ethyl) side chains were carried out to assign experimental IR bands to the vibrational modes, using Gaussian 16 [42] at the B3LYP/6-31G* level of theory and a scaling factor of 0.97. Methyl groups were added at the end of each polymer backbone.

## 4. Conclusions

We studied the molecular orientation of DPP-based low-bandgap polymers in thin films using PM-IRRAS. The investigation of interface properties on substrates with different work functions for polymers 2a and 2c suggests that the choice of the side chains has a minor effect on the interfacial electronic interface structure. Exemplarily, for one polymer, the thickness dependence of the orientation was investigated by IR spectroscopy in transmission. In addition, the absence of a preferred azimuthal orientation could be verified by IR spectroscopy in transmission—a precondition for the orientation analysis by PM-IRRAS. For all polymers, the long axis of the polymer backbone is preferentially oriented parallel to the substrate surface, pointing to a high degree of ordering. In most cases, a preferred face-on orientation was found. The tilt angle of the short axis of the backbone was distinctly higher for a polymer with alkoxy side chains. Thus, the choice of the side chains might be a promising way to tune for face-on and edge-on orientations. This finding demonstrates the crucial role of the side chains for the aggregation properties of DPP-based low band gap polymers, which is in excellent agreement with recent studies with a focus on applications [1,6,7,8]. For example, the high power conversion efficiency in an organic field effect transition is driven to a large extent by the choice of the side chains of the polymers (alkyl or alkoxy) [7].

However, the structure of the backbone also has a significant influence on molecular orientation. The higher degree of ladderization of polymers 1 compared to polymers 2 supports a preferred parallel orientation of the backbone relative to the substrate surface.

## Figures and Tables

**Table 1:** Vibrational frequencies (cm^−1^) together with their assigned vibrations determined from DFT calculations (oop: out of plane, str.: stretching, as.: asymmetric, sym.: symmetric).

1a	1b	2a	2b	2c	Assignment
785	785	781	783	782	CH oop
822	823	822	-	-	CH oop
879	879	-	-	-	CH oop
-	1047	1064	1064	1067	C=C str.
1039	-	1041	-	-	C-O-C str.
1158	1096	1175	1109	1110	CH_2_ rocking
-	1159	-	1160	1160	CH oop
1257	1259	-	-	-	CH str.
-	-	1267	-	-	CH_2_ twisting
-	1180	-	1405	1502	CS, CC as. str.
1360	-	1367	1364	-	CH_2_ wagging
-	1361	-	-	1412	CN, CC as. str.
1437	1441	-	-	-	CC as. str.
-	-	1465	1458	1464	CH_2_ bending
1535	1536	-	-	-	CC as. str.
1567	1569	1564	1557	1556	CN, CC sym. str.
1624	1628	1627	1626	1625	CN, CC sym. str.
1677	1678	1689	1685	1682	C=O str.

**Table 2:** Molecular orientation of the investigated polymers related to the substrate surface, described by angles α and β.

Polymer	1a	1b	2a	2b	2c
α	21°	21°	26°	28°	27°
β	29°	31°	50°	30°	33°

**Table 3:** Molecular weight distributions of the synthesized low band gap (LBG) polymers (M_n_: number average molecular weight, M_w_: weight average molecular weight, DP: degree of polymerization). All polymers were purified via Soxhlet extractions, and the molecular weights of the chloroform fractions were analyzed by gel permeation chromatography (GPC) with tetrahydrofuran (THF) as eluent.

Polymer	M_n_ [kg/mol]	M_w_ [kg/mol]	M_w_/M_n_	DP
**1a**	23.4	111.0	4.74	17
**1b**	16.2	21.7	1.34	12
**2a**	6.6	13.5	2.05	6
**2b**	12.0	20.4	1.71	11
**2c**	7.4	10.3	1.38	6

## Data Availability

The data presented in this study are available on request from the corresponding author.

## Supplementary Materials

The following supporting information can be downloaded at: https://www.mdpi.com/article/10.3390/molecules28186435/s1, Figure S1: Ultraviolet photoelectron spectra for the determination of the ionization potential of polymers 2a and 2c; Figures S2–S5: IR spectra of thin films in transmission as a function of the azimuthal angle for polymers 1b, 2a, 2b, and 2c; Figures S6–S9: IR spectra in transmission of film and pellet for polymers 1b, 2a, 2b, and 2c.
