# Supplementary material for: Influence of Backbone Ladderization and Side Chain Variation on the Orientation of Diketopyrrolopyrrole-Based Donor-Acceptor Copolymers
Source: Molecules. 2023 Sep 5;28(18):6435. doi: 10.3390/molecules28186435 (PMC10535938; doi:10.3390/molecules28186435)
Supplement: Supplementary file 1 [file molecules-28-06435-s001.zip › molecules-2573679-supplementary.pdf]

SUPPLEMENTARY MATERIALS

Influence of backbone ladderization and side chain variation on the orientation of diketopyrrolopyrrole-based donor-acceptor copolymers

Sven Bölke [1], Andreas Früh [1], Florian Trilling [2], Michael Forster [2], Ullrich Scherf [2], Thomas Chassé [1] and Heiko Peisert [1,]*

[1] Institut für Physikalische und Theoretische Chemie, Eberhard Karls Universität Tübingen, Auf der Morgenstelle 18, 72076 Tübingen, Germany;

[2] Makromolekulare Chemie (*buwMakro*) und Wuppertal Center for Smart Materials and Systems (CM@S), Bergische Universität Wuppertal, Gaussstrasse 20, 42119 Wuppertal, Germany;

* Correspondence: heiko.peisert@uni-tuebingen.de

Content

- Ultraviolet photoelectron spectra for the determination of the ionization potential of polymers 2a and 2c
- IR spectra of thin films in transmission as a function of the azimuthal angle for polymers 1b, 2a, 2b and 2c
- IR spectra in transmission of film and pellet for polymers 1b, 2a, 2b and 2c

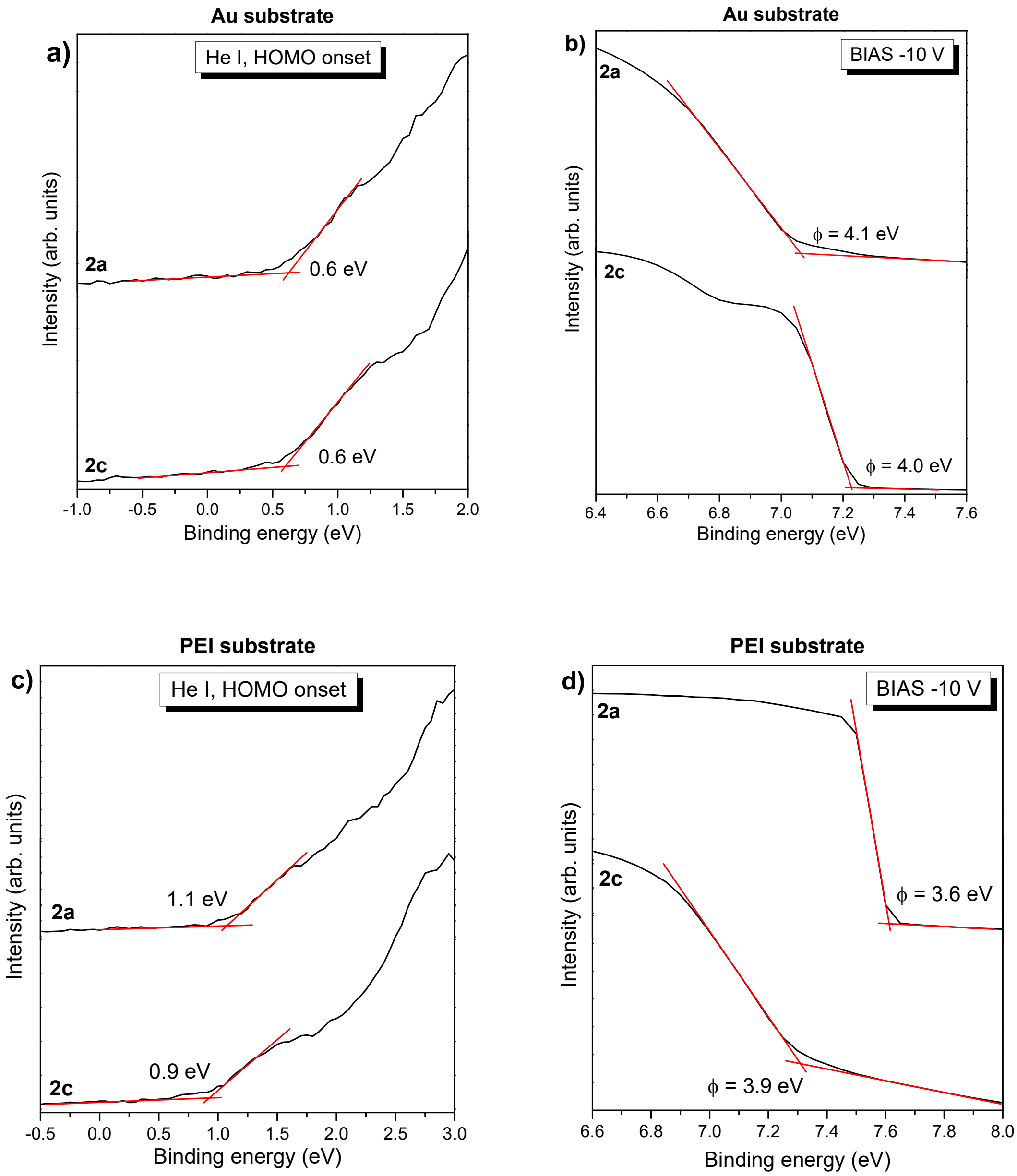

Figure S1. Zoom into the high and low binding energy region of ultraviolet photoelectron spectra (UPS) (excitation: He I radiation) of polymers 2a and 2c for the determination of the HOMO onset E_{HOMO} and the work function Φ. a) and b) are on gold substrate with Φ = 5.2 eV and c) and d) on PEI with Φ = 3.3 eV. The sum of E_{HOMO} and Φ represents the ionization potential IP of the polymers (4.7 ± 0.1 eV).

IR spectra of thin films in transmission as a function of the azimuthal angle for polymers 1b, 2a, 2b and 2c

(Figure S2 – S5)

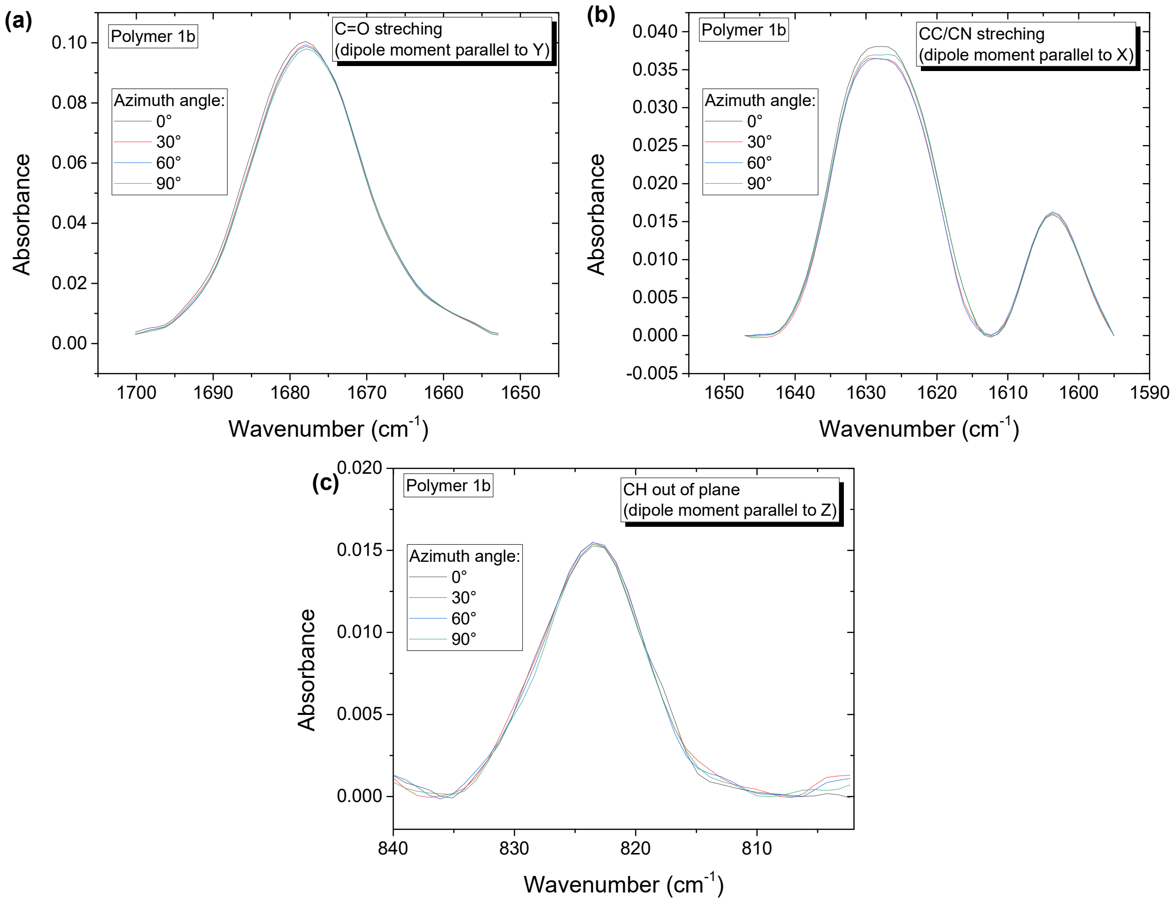

Figure S2. <u>*Polymer 1b:*</u> IR spectra in transmission (polar angle 0°) of an approx. 1200 nm thick film of polymer 1a. (a) C=O stretching vibration b) CN/CC stretching vibration c) CH out-of-plan vibration.

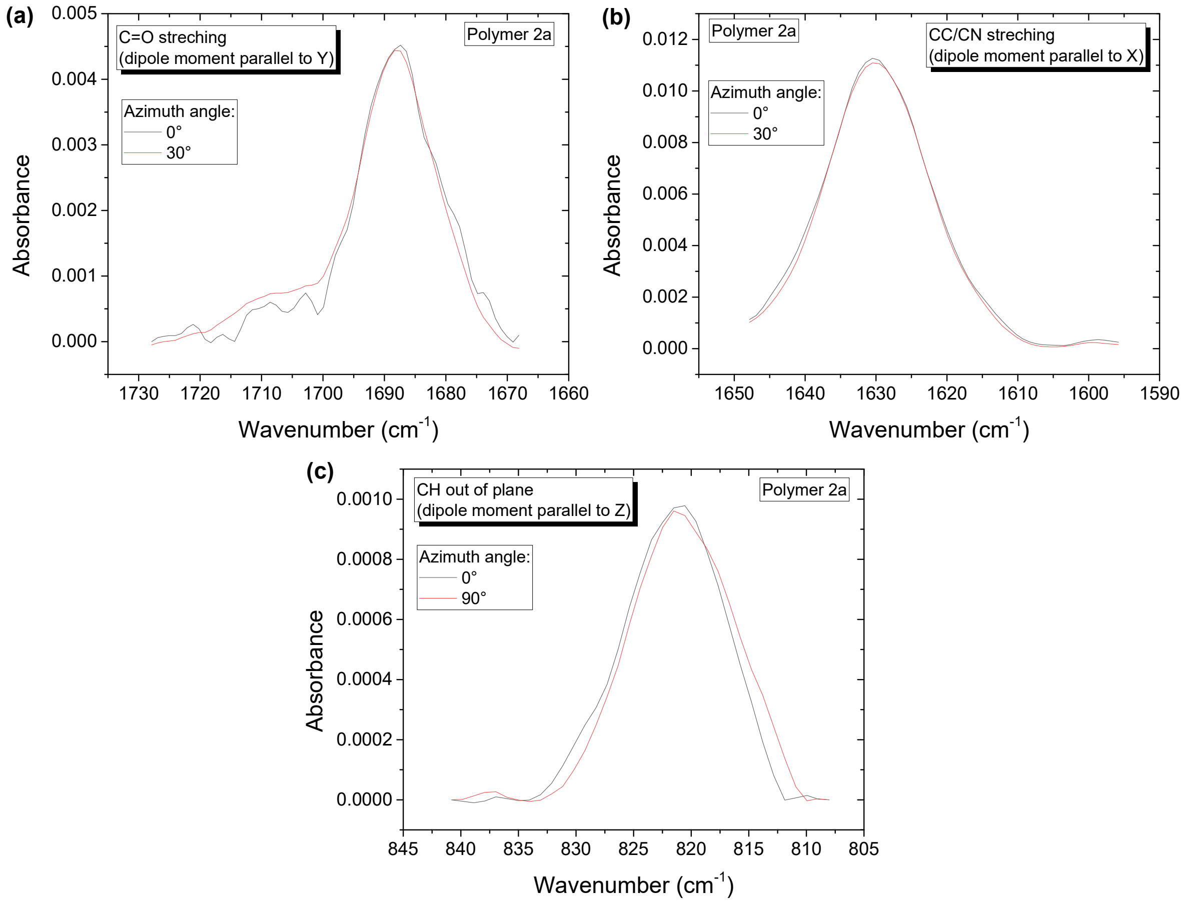

Figure S3. <u>*Polymer 2a:*</u> IR spectra in transmission (polar angle 0°) of an approx. 1200 nm thick film of polymer 1a. (a) C=O stretching vibration b) CN/CC stretching vibration c) CH out-of-plan vibration.

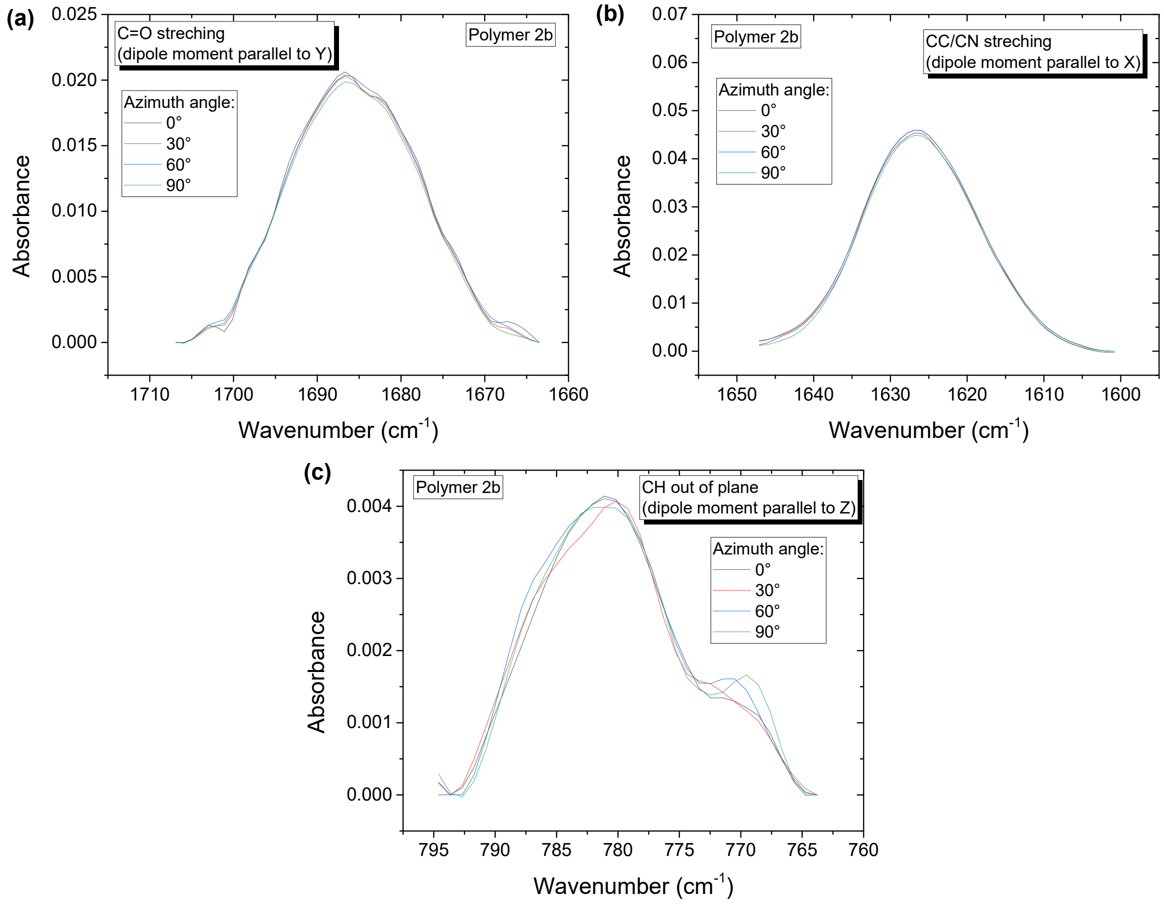

Figure S4. <u>*Polymer 2b:*</u> IR spectra in transmission (polar angle 0°) of an approx. 1200 nm thick film of polymer 1a. (a) C=O stretching vibration b) CN/CC stretching vibration c) CH out-of-plan vibration.

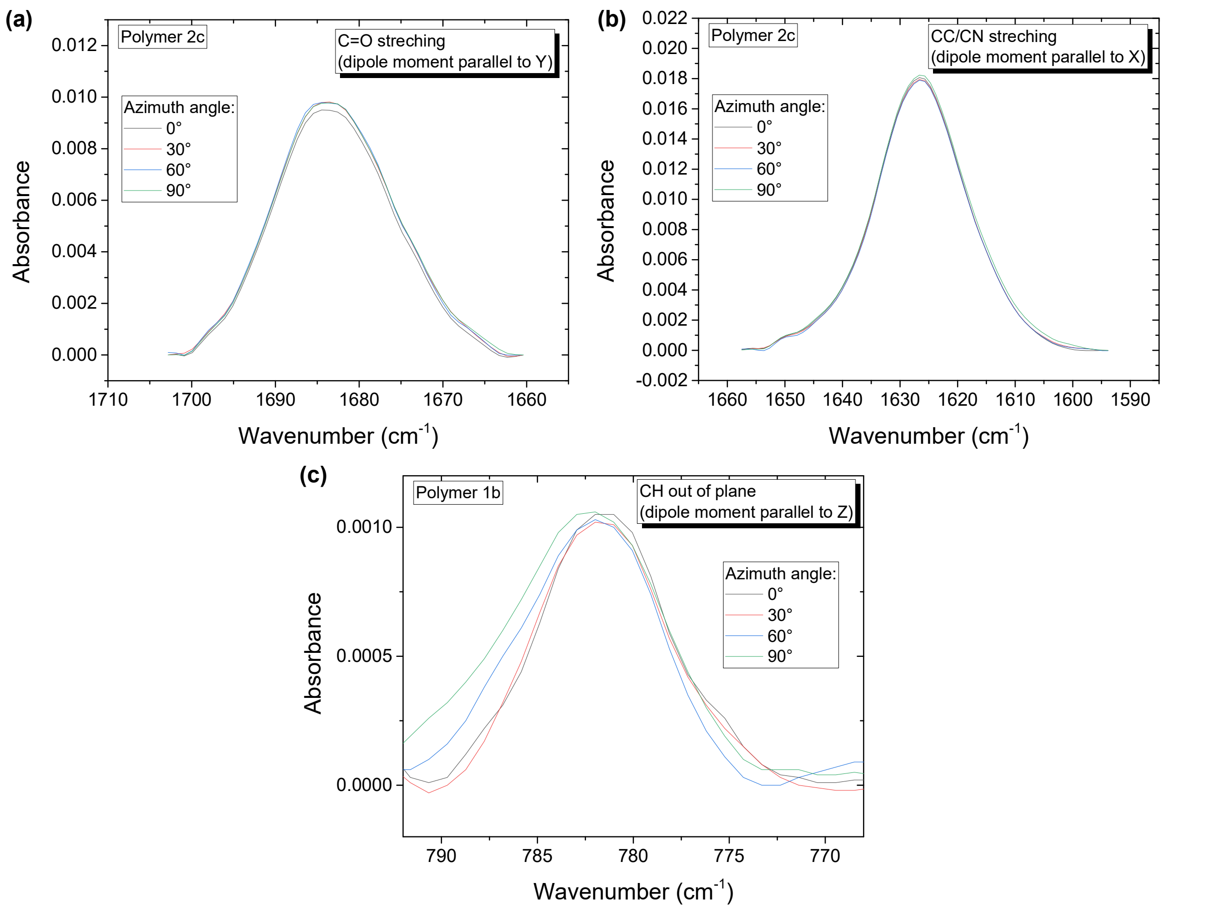

igure S5. *Polymer 2c:* IR spectra in transmission (polar angle 0°) of an approx. 1200 nm thick film of polymer 1a. (a) C=O stretching vibration b) CN/CC stretching vibration c) CH out-of-plan vibration.

Comparison of IR spectra in transmission of films to the reference (pellet) for polymers 1b, 2a, 2b and 2c

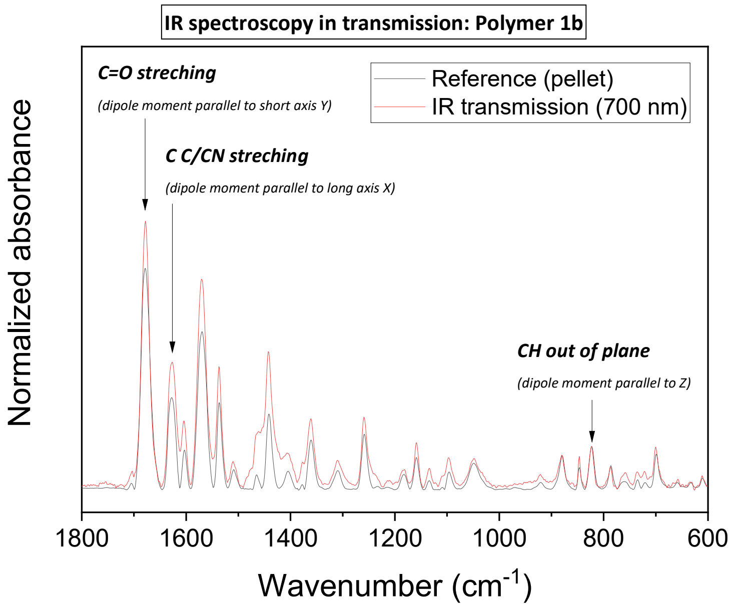

Figure S6. IR spectra in transmission (normal incidence, polymer 1b) in the wavelength region between 600 and 1800 cm^{-1}. Comparison of the transmission spectrum of an approx. 700 nm thick film (red) to the reference of a randomly ordered sample (black, powder pressed in KBr). All spectra are normalized to the out of plane CH vibration at 822 cm^{-1}.

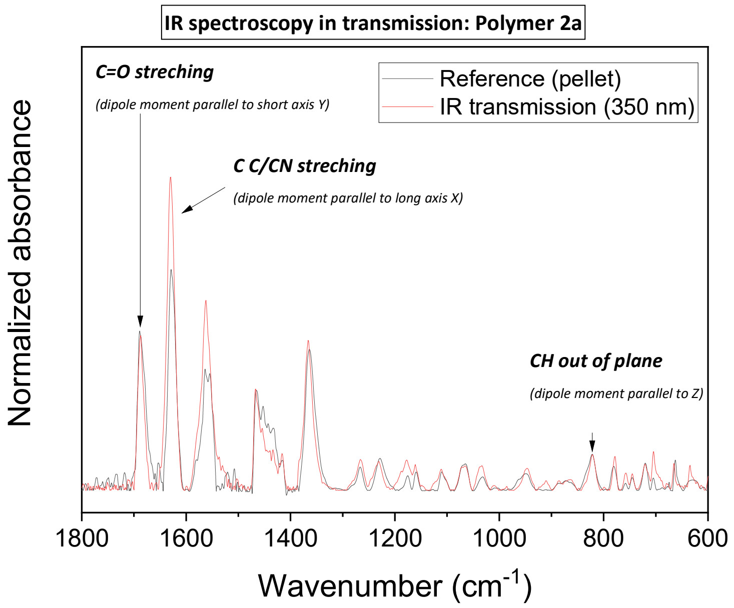

Figure S7. IR spectra in transmission (normal incidence, polymer 2a) in the wavelength region between 600 and 1800 cm^{-1}. Comparison of the transmission spectrum of an approx. 350 nm thick film (red) to the reference of a randomly ordered sample (black, powder pressed in KBr). All spectra are normalized to the out of plane CH vibration at 822 cm^{-1}.

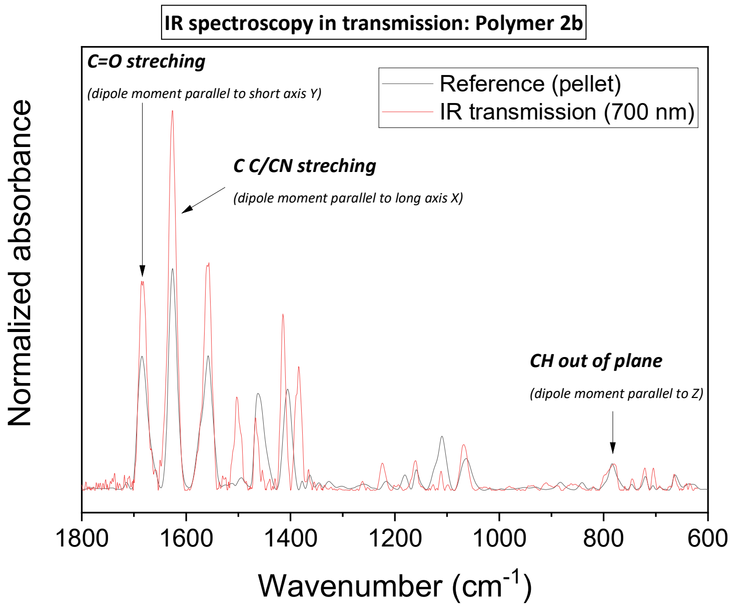

Figure S8. IR spectra in transmission (normal incidence, polymer 2b) in the wavelength region between 600 and 1800 cm^{-1}. Comparison of the transmission spectrum of an approx. 700 nm thick film (red) to the reference of a randomly ordered sample (black, powder pressed in KBr). All spectra are normalized to the out of plane CH vibration at 822 cm^{-1}.

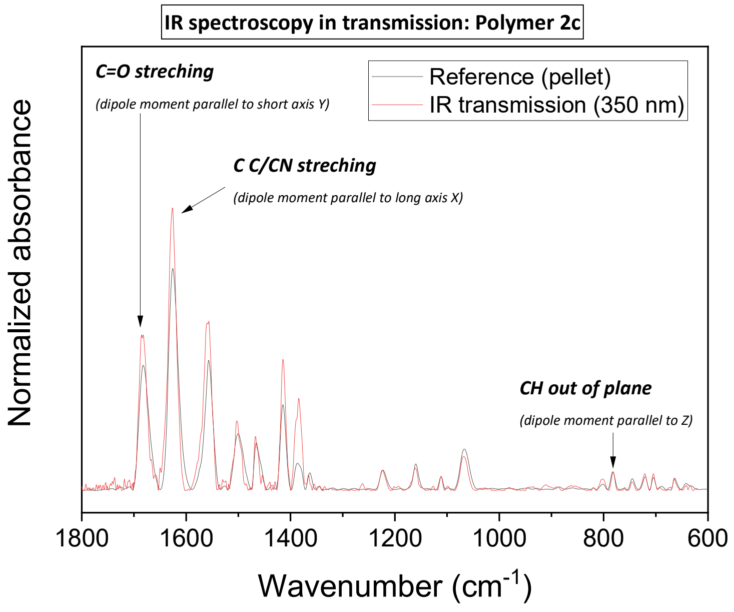

Figure S9. IR spectra in transmission (normal incidence, polymer 2c) in the wavelength region between 600 and 1800 cm^{-1}. Comparison of the transmission spectrum of an approx. 350 nm thick film (red) to the reference of a randomly ordered sample (black, powder pressed in KBr). All spectra are normalized to the out of plane CH vibration at 822 cm^{-1}.
